# Foreign Correspondence

**Published:** 1869-09

**Authors:** 


### Foreign Correspondence.

PARIS, *July 23d, 1869.*

The busy nine months of the medical year are drawing to an end, and the summer vacation approaches. One after another the lectures and clinics have ceased, the dreaded examinations been passed, and the students, taking a long breath for the first time in the year, scatter right and left, abandon Paris and flee to the country, the successful to repose upon their laurels, the plucked to console themselves for their defeat.

By a tacit concurrence in the fitness of things, the patients, the third party in the great triad of professors, students, and sick people, seem to have resolved to postpone their diseases until such time as they can furnish material for the fall lectures. Consumptives recover during the warm weather, the entire group of bronchitis, pneumonias, pleurisies, and emphysemas that throng the hospitals in winter, seem spell-bound by the gracious hand of summer, and so far no cholera has come to supply their place. A lull has fallen upon the field of battle with disease and death, the enemy seems to have drawn off his forces a little, perhaps to recruit, and the soldiers may rest panting on their arms. The battle is so long, so deadly, and must so soon be renewed!

Nevertheless it is a lull, not a cessation of hostilities. Though the amphitheatres be deserted, and the voice of the clinicien be silent in the wards, the academy still holds its séances, and discusses a thousand and one medical subjects with zeal and ability, from which discussions result much entertainment and reputation for the physicians, and occasionally some advantage for the patients.

At the séance of the 9th of July, M. Gavarret read a report on a memoir by Dr. Javal, concerning astigmatism, or ametropia, as it was formerly called. This memoir describes a little apparatus invented for the qualitative and quantitative detection of the remediable form of the disease. The theory of it is based on the following considerations:—

In an eye normally constituted, the surfaces of separation of the different refrangent media are regular, and may be considered as surfaces of revolution around the concentric optic axes. Hence, the power of the natural dioptric apparatus is sensibly the same for all meridians. In other terms, the light, in its passage across the transparent media of a normal eye, obeys the same laws as when traversing an ordinary dioptric apparatus.

It frequently happens, however, that the curve of the surfaces of separation of the transparent media, varies from one meridian to another, so that these surfaces are no longer concentric. This vice of conformation, this asymmetry, occasions the functional difficulty known as astigmatism. An asymmetry in which the variation of the curve increases or diminishes gradually and constantly from one meridian to another, is called regular, and experience and calculation show that this defect may be corrected, and the functional consequences avoided, simply by correcting the asymmetry of the two principal meridians.

The asymmetry may affect at the same time the anterior face of the cornea, and the two faces of the crystalline lens, but it is rare that the two faces of the cornea are involved coincidently with this latter lesion.

M. Javal's apparatus is designed both to detect this defect in the eye, and ascertain the glasses needed to compensate for it. The patient, with his two eyes widely-opened, looks through five-inch convex lenses at a card, upon which are traced two precisely similar dial-plates, which are separated from each other by exactly the same distance as the lenses, and placed exactly opposite the eyes. From the centre of the dial radii, indicating all the hours and half hours, are drawn to the circumference. The angle comprised between any two radii is, therefore, about fifteen degrees. The card is first placed in the focus of the lenses. The patient combines the two images into one; the axes of his eyes are then necessarily parallel. Then, by means of a metallic button, attached to the side of the apparatus, the card is removed as far away as possible from the patient, the images become confused, but remain combined. Then the card is gradually approached towards his eyes until such moment as he can say, "The radii on the dial are all confused and grayish except one; that I see clearly." This indicates, first, that the eye observed is astigmatic; second, the image of the objective card is in the focus of the principal meridian at the minimum curve; third, that the principal meridian at the maximum curve is in the plane of the ray seen clearly.

This ascertained, the oculist passes before the eye to be examined a series of cylindrical divergent lenses, of successively increasing power, from $\frac{1}{96}$ to $\frac{1}{5}$. This series contains twenty different combinations. The apparatus is so disposed that each lens in passing before the eye is in the plane of the principal meridian at the minimum curve, consequently it does not displace the focus of this meridian, while it pushes back the focus of the principal meridian at the maximum curve. When the patient says, "I see all the rays with equal distinctness," the focus of this meridian has been made to retreat sufficiently to coincide with the focus that has not been displaced, and the asymmetry is corrected. The number of the required lens is, therefore, accurately ascertained.

A new method of detecting and measuring the diplopia in ataxia has also been suggested. It is well known that the reason that the two eyes are able to combine into one the double images that come to them from a given object is, that the rays from that object strike upon exactly similar portions of the two retinæ, which are both turned in the same direction. But if the parallelism of the optic axis be destroyed, it is evident that parallel rays will fall upon *dissimilar* regions of the retinæ, and, hence, a double impression will be retained by the brain. The relative position of these two images differs according to the nature of the strabismus. When that is divergent, and the left eye, for instance, is turned towards the left, the rays from an object, directly regarded by the right eye, and seen most distinctly by it, would fall to the right of the left retina, and hence the object would appear placed more to the right than in reality; in other words, its image, crossing that impinging upon the right eye, will be seen on the right side of it. In convergent strabismus, the rays would fall on the left of the left retina, but would pass on inside of those going to the right, and the two images would appear side by side. To make this effect more conspicuous, and, consequently, to detect the incipient degrees of the malady, at a moment when its detection would assist the diagnosis of the ataxia, spectacles have been suggested, of which the right glass should be colored red, and the left blue. The relative position of the colored images are then perceived clearly, at a glance.

The "*Union Medicale*" published the other day an article upon the utility of rhinoscopy and of the naso-pharyngeal douche, invented by Prof. Weber, which, combined, render great service in the diagnosis and treatment of such diseases of the ear as depend upon trouble in the pharynx and posterior nasal cavities, or Eustachian tubes. The rhinoscopic method, as you are aware, consists in placing, at the bottom of the throat, a small mirror, whose polished surface illuminated by a strong light is turned upwards and forwards. This surface, in its turn, throws the light upon the parts to be examined, which are at the same time reflected in the mirror, and thus become visible to the eye of the observer.

Rhinoscopic examination plays an important part in the study of chronic, granular pharyngitis and its complications. In these cases, the physician frequently discovers, near the pavillion of the Eustachian tube, a swelling of the mucous membrane, and so abundant a proliferation of all its elements that the mouth of the tube is completely stopped up by mucosities, which prevent the entrance of air to the middle ear, and interfere with its catheterism. This obstacle can only be detected by the rhinoscopic mirror.

Granular pharyngitis is one of the most frequent chronic diseases of the throat, and is affirmed to be equally common as a complication in diseases of the ear. Sometimes the pavillion of the Eustachian tube is completely enwreathed with granulations. Where it becomes necessary to ascertain the degree of permeability of the tube, the physician may combine the use of the sound with auscultation. This is effected by means of a a tube, putting his ear in communication with that of the patient, when he can directly recognize if air be admitted behind the tympanum.

Weber's douche serves to wash out the nasal fossæ, the posterior nostrils, and the Eustachian tubes, and to inject medicated fluids upon them. It differs from the means hitherto employed, inasmuch as it enables the physician to pass a gentle and continuous current over the diseased parts. The instrument is furnished with an extremity exactly the size of the nostril into which it is inserted, so that the liquid cannot run backwards. This liquid, gently pushed by means of a syringe, fills the nasal cavity of one side, passes into the upper part of the pharynx, where it bathes the mouths of the Eustachian tubes, and, finally, runs out at the nostril of the other side. It will not go down the throat, for Weber has proved by experiment that when the nostrils are entirely closed the soft palate is raised and closes the bottom of the pharynx. It is to be supposed, therefore, that at the beginning of the operation, before the liquid has reached the second nostril, this would need to be stopped up by something corresponding to the extremity of the instrument which occupies the first; but this is not specified in the " *Union Medicale.*"

While speaking of diseases of the pharynx, it would not be out of place to notice a remarkable case of pharyngeal tumor that occurred in the service of M. Richet, at the Hotel Dieu. This tumor was enormous, and developed at first in the soft palate, seemed to perfectly answer the description of tumors that are developed in this region, from a hypertrophy of its glandular elements. It was ulcerated, and rendered deglutition and even respiration extremely painful. M. Richet resolved to operate, but bethought him first of trying the effects of sublimate and iodide of potassium. This treatment was entirely successful; the tumor rapidly diminished in size, the ulceration healed, and, by this time, the patient is considered about well.

At the Imperial Society of Surgery, the other day, M. Broca presented a Peruvian skull, found by Squier in an old tomb of the Incas, in the valley of Yucay, near Cuzco, and belonging to a date anterior to the time of Cortez. The interest of the skull to surgeons lay in the fact that it had been trepanned! The perforation was situated on the left side of the frontal bone; and from the appearance of the surrounding osseous tissue, no doubt remained that the operation had been performed on a living person. M. Nelaton estimated that the patient should have survived the trepanning about eight days.

The bone presented no trace of fracture, and after examination of that part of the inner table which adjoined the perforation, the surgeons unanimously concluded that the operation had been undertaken on account of some internal lesion, an enterprise which indicates rather advanced notions in theoretical surgery. But the act did not come up to the theory, since it was evident from the appearance of the square opening, with straight and regular edges, that the surgeon had been provided with no instrument specially appropriate to the purpose, but had operated with some rude and ordinary chisel.

At the last séance of the Biological Society, M. Charcot read a paper upon certain symptoms marking the début of cerebral hemorrhage. This distinguished physician declared, it was an error to suppose that the paralyzed side of the body is permanently colder than the other. The lowering of the temperature really occurs, but is but momentary, and a reaction speedily sets in, which raises the temperature of the paralyzed part. It is extremely remarkable that in respect to their cadaveric rigidity, the paralyzed muscles present almost the same characteristics as those observed in the muscles of persons who have been struck with lightning. That is to say, they enter with unusual promptitude upon this taste, which is, however, but slightly intense, and of short duration.

Now, Brown-Séquard has observed that in paralysis of the vaso-motor nerves, the vitality of the parts seemed to be exalted, electric excitability greater, and the cadaveric rigidity more tardy, more intense, and of longer duration. In these respects, therefore, a real antagonism seems to exist between the sympathetic and the cerebro-spinal nervous systems. But Charcot's other observations concerning the rise of temperature in paralyzed limbs seems rather to coincide with these remarks of Séquard's, and with Claude Bernard's experiments, by which section of the sympathetic nerve was followed by precisely similar effects.

A patient has just left the wards of M. Hérard, at Lariboissiere, who has excited much interest as the subject of that rather rare disease glosso-labial paralysis. The patient was a man about 50, of a robust constitution, large, strong built, though not fleshy, well nourished, in good health up to the time of the accidents in question. He entered the wards on the 20th of May, and gave the following account of himself:—Four or five days previous, on waking up in the night, he discovered that he had completely lost the power of speech. He declares that he experienced no unusual sensation; yet this seems scarcely probable, since, as he was alone, there seems no reason why he should have attempted to speak, and thus discovered his incapacity, unless prompted by some uneasiness, however vague. Much frightened, he contrived to summon assistance and send for a physician; and the latter, who arrived the next morning, put a blister to the nape of his neck. In a day or two, the aphasia was diminished, and the patient could mumble out certain sounds; but as the concomitant symptoms to be immediately described still persisted, and the power of speech was in anything but a satisfactory condition, the patient, about the 5th day of the disease, entered the Hospital, where the following observation was made of his case:—

The intelligence, which seems to be naturally vivacious, is perfectly intact. There is not the slightest perversion of motility or sensibility in any of the limbs, and the patient suffers no pain, either in the head or in any other part of the body. No perversion of any of the special senses. The face, in repose, presents nothing of note, except that the left corner of the mouth falls a little, and the right is drawn. This difference persists during the act of laughing, but there is then no general deformity, as in facial hemiplegia. The sensibility of the entire face, of the lips, mouth, and tongue, is intact, as also the sense of taste.

When the patient attempts to speak, he uses most violent efforts, twisting first the lips, then the entire head, and often suddenly embracing the person he addresses, as if to convey by touch a meaning so imperfectly expressed by his tongue. There is no fibrillar trembling of the lips, as in progressive paralysis, and there is a great deal more sound and effort at articulation than in true aphasia. But the difficulty seems to lie in the closing and adjustment of the lips; hence, all sounds in which they are especially called into play, as the utterence of the sabialis, and most of the separate vowels, are quite impossible for the patient. He mumbles between his teeth and in his throat, and his difficult speech is affected with a curious precipitation and irregularity of rythm; he struggles desperately with a few words, then, as if the vocal apparatus had been forced into momentary working order, and he dreaded to lose the opportunity, he utters a number of words in rapid succession, and stops abruptly. It is precisely as if the voice slid rapidly down hill and came to a sudden bump at the end!

The difficulty of articulation is increased by the coincident paralysis of the tongue, over which the patient has almost completely lost control, and is scarcely able to move it in any direction, especially towards the right. There is no deviation.

Besides the tongue and the orbicularis, the buccinator muscles of both sides are partially paralyzed. The patient is utterly unable to distend them in the actions of whistling, smoking, or blowing upon a trumpet. A more serious consequence of this paralysis is, that the food is with difficulty retained in place during mastication, but falls in front of the gums, from whence the patient is obliged to extract it with his fingers, and push it back towards the pharynx.

The uvula and soft palate are unaffected, and hence there is no change in the timbre of the voice, which usually becomes nasal in case of their paralysis. Deglution is facile after mastication is once accomplished.

Such is the assemblage of symptoms presented by the patient, and it is evident at a glance, how difficult it is to explain them. The nerves principally involved are, of course, the hypoglossals and the buccinator and orbicular branches of both the facials, with a preponderance towards the left. Hence, the lesion has not spread according to contiguity of the nervous roots, but according to the connection of nerve functions, which of itself is a remarkable circumstance. The nature of the lesion is most difficult to determine, or even suspect, especially for the case in question, which was anomalous for the suddenness with which the accidents were produced. In the other cases that have been reported, especially by Trousseau, and more recently by M. Alfred Fournier, the disease has always invaded the parts with most insidious slowness; and the few autopsies that have been made have generally revealed a scleriasis or atrophy of the roots of the hypoglossal nerves, frequently extending to the glosso-pharyngial, and even involving the pneumo-gastric. In these cases, however, the soft palate and uvula were always paralyzed. But, with Hérard's patient, the paralysis was produced as suddenly as that consecutive to a cerebral hemorrhage; and the rapidity with which, at the beginning, an improvement took place (improvement followed, as we shall presently see, by a perfectly stationary condition) is also precisely such as occurs when the fluid portion of the effusion is quickly absorbed, and the fibrinous coagulum left for a slower process. But it is almost certain that the most limited effusion at the root of any of the nerves affected—that is to say, in the fourth ventricle, or in the medulla oblongata—would have been followed by instant death. Moreover, in so narrow a space, several other nerves could hardly fail to be affected, which were evidently untouched —the glosso-pharyngial, pneumo-gastric, and spinal accessory. Finally the facial nerve was not paralyzed throughout all its extent, but merely in the regions indicated above. The play of the nostrils was quite free. It seems most probable, therefore, that if any hemorrhage is to be blamed, it must have occurred in the brain, in some part destined to preside over articulation, and to that extent would be allied to aphasia, and M. Brœa's third, left convolution of the hemispheres. But, as we have already seen, the character of the tortured and twisted articulation differed much from the almost absolute muteness of aphasia. Moreover, in that disease there is no deviation of the mouth, or paralysis of the tongue or buccinator.

The prognosis of this disease is as curious, I may say as unexpected, as its diagnosis. It is, according to Trousseau and to Fournier, *almost invariably fatal.* Fournier has analyzed the proximate causes of death, and shown that they all depend on the difficulties in deglutition. In Herard's patient, these difficulties were comparatively slight, though productive of sufficient inconvenience. But when, in addition to the paralysis of the buccinators, the reflex action of the tongue is destroyed, so that it cannot arch against the palatine vault and at once propel the alimentary bolus towards the pharynx and shut off its reflux towards the orifice; when the soft palate no longer rises to close the nasal cavities, and the uvula no longer directs the closure of the epiglottis, then the food falls out of the mouth, into the larynx, into the nose, thus producing the most violent strangulation; it frequently causes ulcerations from long sojourn in the buccal cavity, and so insufficient a quantity is swallowed that the patient actually starves to death.

With the patient at Lariboissiere, the affair turned out differently. Scarified cups were applied to the neck on the first day, the second, a blister, the third, a purgative clyster was administered. After that, a blister was kept in permanence at the nape of the neck, for two or three weeks. For the first week, the improvement in his condition that had already begun before his entrance continued. By the 27th, the patient could speak so as to be understood, though with difficulty. He no longer embraced the persons he addressed. He could also smoke his pipe, but could not whistle. The food no longer fell in front of the gums. He could easily turn the tongue to the left, but not at all to the right, and only with difficulty upwards. The effort deformed the right labial commissure. The general health continued perfectly good. At this point, the patient remained perfectly stationary till the 20th of June. About this time he began to be treated with sulphur baths, three times a week, and electricity every day, the current being directed to the nape of the neck and the muscles of the mouth. After this, he could talk much longer at a time, although the difficult articulation continued. The patient left the Hospital the 25th of July, in that same state.

Leaving further comments on this case to persons of greater sagacity than myself, I must find space to mention a patient in the wards of M. Bouilland, at La Charité, who is in the seventh year of a remarkable muscular atrophy, affecting the trunk and limbs. I was fortunate enough to be present in the ward one day during a visit made by M. Duchenne (who is quite famous in connection with this disease, as well as the *ataxic locomotrice* that bears his name), who exhibited and explained the case, and applied it to the demonstration of certain theories of his own. I will mention two of them.

In the first place, it is to be noticed that the scapular muscles are the only ones that are not wasted almost to nothing, and in complete fatty degeneration. The deltoid is a mass of fat. But, nevertheless, the patient is still able to raise his arm to a level with the shoulder; and when M. Duchenne supplied the place of the atrophied grand serratus, by forcible pressure upon the shoulder-blade, so as to fix it against the ribs, the patient could even place his hand on the top of his head, "which proves," observed M. Duchenne, "that the supra-spinous muscle, which alone is left to effect this action, is far more powerful than generally supposed, and in spite of the nearness of its insertion to the articulation, and can of itself supplement the deltoid."

But the most remarkable phenomena are those connected with the respiration. The intercostal muscles are almost gone (their place being supplied by fat), and the shape of the chest entirely changed. The antero-posterior diameter is much shortened, and the transversal lengthened, so that the chest is notably flattened and broadened, and resembled, by actual measurement, the following figure:—The inferior extremity of the sternum is directed backwards. The scaleni, serrati, pectoral, and other muscles devoted to the elevation of the ribs being atrophied, these do not rise during inspiration: the chest remains perfectly motionless, and the breathing is effected entirely by means of the diaphragm. As the abdominal muscles are completely flaccid, they offer no resistance to the descent of the diaphragm, or the projection of the abdominal viscera, and a prodigious eventration occurs at each inspiration. If the hand be forcibly laid upon the abdomen, so as to resist the projection of the viscera, the force of the diaphragm is expended on the false ribs, which are *drawn inwards;* exactly the contrary to what occurs in normal adult inspiration. Beau and Massiat have, however, observed this phenomenon in children, when their breathing is violent or agitated.

From the transformation in the shape of the thorax, M. Duchenne drew the following inference:—"Since the cavity was diminished coincidently with an atrophy of the intercostal muscles, it follows that the office of these muscles is not to diminish the capacity of the chest, since that diminution is effected at a moment when they are impotent; and since the cavity is diminished in expiration, it follows that these are not expiratory muscles, as maintained by Beau and Massiat, but perpetual antagonists of the expiratory force, which triumphs the instant they are *hors de combat;* in other words, that the intercostals are muscles of inspiration.

But, in the humble opinion of your correspondent, M. Duchenne does not prove his case, at least with these facts; but his own words turn against him. For, supposing that the intercostals be really "the constant antagonists of the expiratory force," this implies that they act continually, and not intermittently, as they must do, if called upon simply to contract during inspiration. Not their *contraction* but their *tonicity* is required to counterbalance the elasticity of the chest, which is, like the lungs, in a state of forced extention, and continually tends to fold upon itself, bending at the angles of the ribs. When this tonicity is destroyed, the chest collapses, as proved by the remarkable case in question, and; hence, it is true that such collapse is due to the atrophy of the intercostal muscles, but not for the reason assigned by M. Duchenne.

In surgery, I have only to report this week, as belonging to my personal observation, an operation performed by Professor Jarjavay, at Beaujon. It was the excision of a fibrous tumor, developed in the abdominal parietis of a woman, of good constitution, about 35 years old. The tumor was broad and rather flat, about the size of the palm of the patient's hand, and had existed for two years without causing much uneasiness. But, latterly, it had begun to increase, and as it was evidently extra peritonial, and apparently attached to the crest of the iliac bone, the Professor resolved to operate, expecting either to be able to ennucleate the tumor, or to find it sufficiently pediculated, to render its excision facile. In both hopes he was disappointed. The tumor was firmly attached by a broad base to the peritoneum itself; and the surgeon having ennucleated it to this point, removed the bulk of the mass in successive slices, leaving, however, one slice adherent to the peritoneum, which, therefore, was uninjured. The patient is doing well.

The *Bulletin de Therapeutique* reports a case of successful treatment of acute mania by digitalis. A boy of 14 was received at the Hotel Dieu, in the service of M. Vernois, in a condition of extremely incoherent delirium, and with all his limbs affected with disordered movements, very much like those of chorea. The diagnosis between a veritable mania, with extreme agitation, or a chorea accompanied by delirium, remained doubtful. On his entrance, the patient had been put into a strait-jacket; but M. Isambert coming to supply the place of M. Vernois ordered the strait-jacket to be taken off, and 30 drops of tinctue of digitalis administered every day. From the commencement of this treatment, the vociferations and disordered movements ceased, and there remained a low, muttering delirium. Inquiry into the boy's antecedents then disclosed the fact, that for the last year he had been in a condition of real lypemania, of which the mania was an acute crisis. It, therefore, became unquestionable that digitalis had been able to exercise a valuable influence in calming acute mania.

Dr. Savignac inserts two formulæ in the *Bulletin*. The first is directed against cholera:—

Ether, 64 grains (4 grms.)

Ext. Rhat., 64 grains (4 grms.)

Syrup opii, 480 grains (30 grms.)

Aqu. Menth., } āā 960 grains (60 grms.)

Aqu. Mellis, }

Mix the aqu. menth. and aqu. mel. together; dissolve in them the ext. rhat.; add the syrup opii, and, finally, the ether. A desert spoonful every quarter of an hour, until the severity of the symptoms are abated, then, it may be given at longer intervals.

The second formula is for dysmenorrhœa:—

Acet. Ammon., 80 grains (5 grms.)
Aq. Orang. Flor., } 640 grains (40 grms.)
Aq. Mel., } 1280 grains (80 grms.)
Syrup Saffron, 480 grains (30 grms.)

Dose: a desert spoonful. In very severe cases, add 15–20 drops of Sydenham's laudanum.

